# Phenotypic characterization of *Plasmodium berghei* responsive CD8+ T cells after immunization with live sporozoites under chloroquine cover

**DOI:** 10.1186/1475-2875-13-92

**Published:** 2014-03-12

**Authors:** Clara Brando, Jason H Richardson, Jittawadee Murphy, Christian F Ockenhouse, Edwin Kamau

**Affiliations:** 1 Military Malaria Research Program, Malaria Vaccine Branch, Walter Reed Army Institute of Research, 503 Robert Grant Ave, Silver Spring, MD 20910, USA; 2 Entomology Branch, Walter Reed Army Institute of Research, 503 Robert Grant Ave, Silver Spring, MD 20910, USA

## Abstract

**Background:**

An effective malaria vaccine remains elusive. The most effective experimental vaccines confer only limited and short-lived protection despite production of protective antibodies. However, immunization with irradiated sporozoites, or with live sporozoites under chloroquine cover, has resulted in long-term protection apparently due to the generation of protective CD8+ T cells. The nature and function of these protective CD8+ T cells has not been elucidated. In the current study, the phenotype of CD8+ T cells generated after immunization of C57BL/6 mice with live *Plasmodium berghei* sporozoites under chloroquine cover was investigated.

**Methods:**

Female C57BL/6 mice, C57BL/6 mice B2 macroglobulin −/− [KO], or invariant chain−/− [Ic KO] [6–8 weeks old] were immunized with *P. berghei* sporozoites and treated daily with 800 μg/mouse of chloroquine for nine days. This procedure of immunization is referred to as “infection/cure”. Mice were challenged by inoculating intravenously 1,000 infectious sporozoites. Appearance of parasitaemia was monitored by Giemsa-stained blood smears.

**Results:**

By use of MHC I and MHC II deficient animals, results indicate that CD8+ T cells are necessary for full protection and that production of protective antibodies is either CD4+ T helper cells dependent and/or lymphokines produced by CD4 cells contribute to the protection directly or by helping CD8+ T cells. Further, the phenotype of infection/cure *P. berghei* responsive CD8+ T cells was determined to be KLRG1^high^ CD27^low^ CD44^high^ and CD62L^low^.

**Conclusion:**

The KLRG1^high^ CD27^low^ CD44^high^ and CD62L^low^ phenotype of CD8+ T cells is associated with protection and should be investigated further as a candidate correlate of protection.

## Background

While reduction in malaria cases has been reported in many countries, malaria remains among the world’s most prevalent and fatal infectious disease. In 2011, it was estimated there were 216 million malaria episodes and 655,000 malaria deaths [1]. With the majority of the deaths occurring in children less than five years of age, and with almost half of the world population at risk, an effective vaccine against malaria is urgently needed [2].

Natural exposures to malaria infections do not immediately induce immunity leaving infants and young children in endemic areas susceptible to multiple episodes of the disease. Eventually, partial immunity is acquired in older children and adults, affording them protection against clinical symptoms and/or severe disease. However, protection is not sterile [3] and the immune responses to *Plasmodium* parasites are short-lived [4]. This is attributed to short half-life of protective antibodies [4] and to a cellular response [5-7] too weak to grant protection [8-11]. Sterile protection has however been achieved experimentally in both animal models of malaria and in malaria-naive humans immunized with whole live sporozoites [12].

Intravenous administration of irradiated sporozoites renders long-lasting protection [13-17] by a mechanism mediated largely by CD8+ T cells [18-23]. Although there is data in favour of other mechanisms [24] and different mice strains show different susceptibility to malaria [25,26], CD8+ T cell remain the main player in this model of protection. Evidence of protection conferred by immunization with sporozoites under chloroquine (CQ) cover was initially demonstrated in mice and rats using *Plasmodium berghei*[27,28]. More recently, infection under CQ cover has also been shown to induce long-lasting protection in malaria-naïve human [29,30] and murine models [31,32]. In these models, CD8+ T cells appear to play an important role in protection [24,29-32]. Taken together, these data suggest that while natural exposure elicits weakly protective humoral and cellular immunity, strategies such as inoculation of irradiated sporozoites or viable sporozoites under CQ cover induce CD8+ T cells which, alone or in combination with antibodies, provide long-lasting protection. A better understanding of the mechanics of these protective cells is fundamental to vaccine development efforts.

In this study, *P. berghei* murine model was used to characterize the phenotype of CD8+ T cells generated under CQ cover and to associate these phenotypes with protection from a lethal challenge.

## Methods

### Mice

Female C57BL/6 mice, C57BL/6 mice B2 macroglobulin −/− (KO), or invariant chain−/− (Ic KO) (6–8 weeks old) were purchased from the Jackson Laboratory (Bar Harbor, ME). These animals were housed at the Walter Reed Army Institute of Research (WRAIR) animal facility and handled according to institutional guidelines. All procedures were reviewed and approved by the WRAIR Animal Care and Use Committee (IACUC) and were performed in a facility accredited by the Association for Assessment and Accreditation of Laboratory Animal Care International. Euthanasia methods were compliant with the approved IACUC protocol, with frequent supervision by the institute’s veterinary team. After the animals were challenged, strict humane endpoints criteria were adhered to by frequently monitoring the health of the animals in order to avoid or terminate unrelieved pain and/or distress. Animals positive for parasitaemia for two consecutive days were euthanized before showing distress and/or pain by inhalation of CO_2_ from a pressurized tank in a chamber. This was followed by cervical dislocation prior to disposal of the dead mice.

### Sporozoites

Sporozoites (Spz) from *P. berghei* ANKA strain were extracted from salivary glands of *Anopheles stephensi* mosquitoes 16–21 days post blood meal as previously described [19]. Mosquitoes were maintained in the WRAIR Entomology Branch Insectary facilities.

### Reagents, antibodies and chemicals

Cell media and CFSE were purchased from Life Technologies (Carlsbad, CA). Purified antibodies or fluorochrome labeled antibodies were purchased from eBioscience (San Diego, CA). Brefeldine A, Giemsa stain and CQ were purchased from Sigma (St. Louis, MO).

### Immunization and treatment

Experimental animals were inoculated two or three times intravenously with 10,000 or 20,000 Spz (in 100 ul PBS), ten days apart and treated daily with 800 μg/mouse (100 ul in PBS) of CQ, administered intraperitoneally (i.p.), starting from the day of the first inoculation until nine days post last inoculation. This procedure of immunization is referred to as “infection/cure”. For the CQ control group, the same regimen of CQ as that of the experimental group was administered with the exception that these animals were not inoculated with Spz. The absolute control group received PBS only.

### Challenge and assessment of protection

For challenge experiments, mice were inoculated intravenously with 1000 infectious Spz. Challenge was performed at two weeks post suspension of CQ treatment. Appearance of parasitaemia was monitored by Giemsa-stained blood smears. Animals free of parasitaemia for two weeks were considered protected.

### Lymphocyte preparation

Mice were euthanized by CO_2_ inhalation as described above. Livers and spleens were removed, and livers were perfused with 10 ml PBS. Single cell suspensions of lymphocytes were made from both organs (liver infiltrating lymphocytes and splenocytes). Cells were re-suspended in PBS and used for analysis or transfer. Peripheral blood for blood lymphocyte analysis was collected from the tail vein in a heparinized vial.

### Serum and splenocyte transfer

Recipient mice in transfer experiments received either 500 μL/mouse of undiluted serum intraperitoneally, 20 × 10^^6^ splenocytes re-suspended in 500 μL of PBS by injection into the tail vein, or a combination of both.

### Flow cytometry analysis

Analysis was performed with an LSR II cytometer (Beckon Dickinson San Diego, CA) and data were analysed with Flow-Jo software (BD). The panel of fluorochrome conjugated antibodies for flow cytometry included: CD3- PrcP, CD4-Pacific Blue, CD8-V500 or Pacific Orange, CD45RB or CD44- Alexa fluoro −700, CD27-APC, CD127 –PE, KLRG-1 FITC. When the intracellular cytokine IFNγ was tested, the panel used was: CD8-PO, KLRG-1-FITC, CD62-PrCPcy5.5, CD44 Alexa-700, CD27-APC, IFN- γ-PE. In all assays UV-viability dye was included. Flow cytometry antibodies were purchased from Life Technologies (Carlsbad, CA).

### Abbreviation for fluorochrome

Allophycocyanin (APC), Pyridine-chlorophyll proteins-Cy-5 (PrCP-Cy5), Phycoerythrin (PE), Fluorescein (FITC). Antibodies are indicated by the marker recognized-fluorochrome, e.g. anti CD3 Fluorescein-conjugated was CD3-FITC.

## Results

### Three cycles of infection/cure results in protection

**Figure 1:**
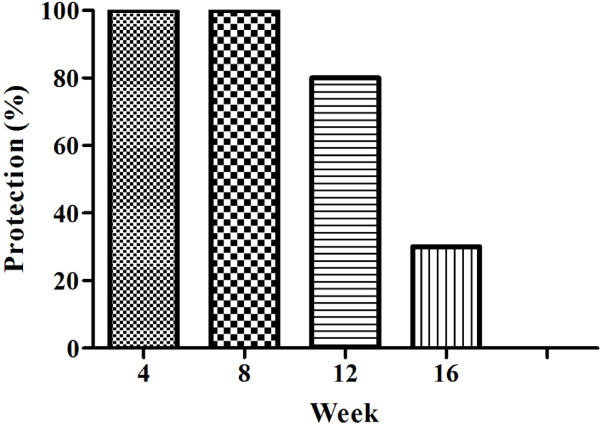
**Infection with sporozoites under chloroquine cover induces long lasting protection.** Infection with sporozoites under CQ cover induces long lasting protection. Three groups of ten mice each were inoculated three times, two weeks apart with 20,000 Spz. Each group of animals was challenged once with 1,000 sporozoites-at week 4, 8, 12 and 16 post the last inoculation as shown. For each group five naïve mice were used as infectivity controls. Appearance of parasitaemia was followed by Giemsa staining of blood smears.

To establish the infection and cure model of C57BL/6 mice, animals were inoculated with infective Spz in the presence of CQ as described in the method section, referred to as infection/cure. Different dose and frequency regimens of infection/cure were compared (Table 1). Three groups of animals were challenged 4, 8 or 16 weeks post the last inoculation. Animals whose blood was negative for parasitaemia at 14 days post challenge were declared protected. One hundred percent of the animals that received three-inoculations of 20,000 Spz remained fully protected when challenged eight weeks post third inoculation, whereas those challenged 16 weeks post third inoculation exhibited only 40% protection (Figure 1). All animals treated with CQ (but not inoculated with Spz) and challenged two weeks post suspension of the CQ treatment became parasitaemia confirming a lack of a residual protective effect of CQ following suspension of the drug. This data indicates infection/cure immunization with three, 20,000 Spz inoculations resulted in protection.

**Table 1:** Immunization regimen with Spz used for infection/cure of mice (10 animals/group)

**Spz (dose)**	**Immunizations (n)**	**Protection (%)**
10,000	3	80
20,000	2	40
20,000	3	100

### Protection requires both CD8+ T cells and antibodies

**Figure 2:**
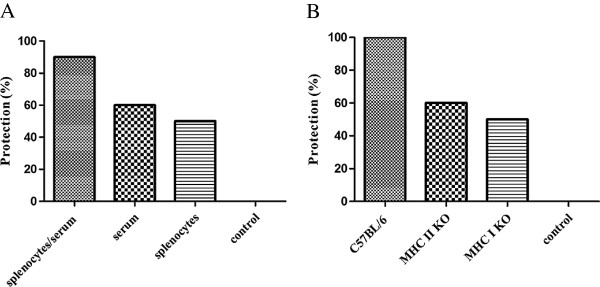
**CD4+ T cells and CD8+ T cells are required for full protection. A)** Infection/cure animals were sacrificed two weeks post suspension of CQ treatment. Splenocytes and/or serum from immunized or non-immunized animals were harvested and transferred into naïve C57BL/6 mice. Groups of ten recipient mice received 2 × 10^7^ cells, 100 μL serum or a combination of both. Twenty-four hours later animals were challenged with 1000 Spz and appearance of parasitaemia was monitored for two weeks (representative of two experiments). **B)** Ten C57BL/6 wild type, or Ic−/−(MHC II KO), or β2 macroglobulin −/− (MHC I KO) were infected/cured under CQ cover as described, group of animals received CQ alone as a control ( results from one experiment). Two weeks post suspension of the CQ treatment animals were challenged with 1,000 Spz and the appearance of parasitaemia monitored for two weeks.

To investigate the relative role played by the cellular and humoral immune response in protection from the challenge, spleens and serum were collected from infection/cure animals two weeks after suspension of CQ treatment and transferred into naïve animals. Groups of 10 naïve C57BL/6 mice were given either splenocytes, serum or both as described in the methods section. Recipient animals were challenged 24 h post-transfer and parasitaemia monitored for two weeks. Ninety percent of the animals treated with both splenocytes and immune serum were protected. Animals that received splenocytes or immune serum alone showed 50% and 60% protection respectively (Figure 2A). This data points to an additive protective effect of cellular and humoral immunity. Having demonstrated that cellular immunity was required to grant 100% protection, the role of CD4+ T cells and CD8+ T cells in protection using Ic−/− (MHC II KO, CD4+ cell deficient) and β2 microglobulin−/− (MHC I KO, CD8 + cell deficient) mice was investigated. Immunized or non-immunized groups of 10 C57BL/6, MHC II KO or MHC I KO mice were challenged two weeks after suspension of the CQ treatment. As expected, wild type C57BL/6 mice were fully protected whereas MHC II KO and MHC I KO animals were only partially protected (Figure 2B). These results indicate that CD8+ T cells are necessary for full protection and that production of protective antibodies is either CD4+ T helper cells dependent and/or lymphokines produced by CD4 cells contribute to the protection directly or by helping CD8+ T cells.

### The phenotype of infection/cure *P. berghei* responsive CD8+ T cells is KLRG1^high^ CD27^low^ CD44^high^ and CD62L^low^

**Figure 3:**
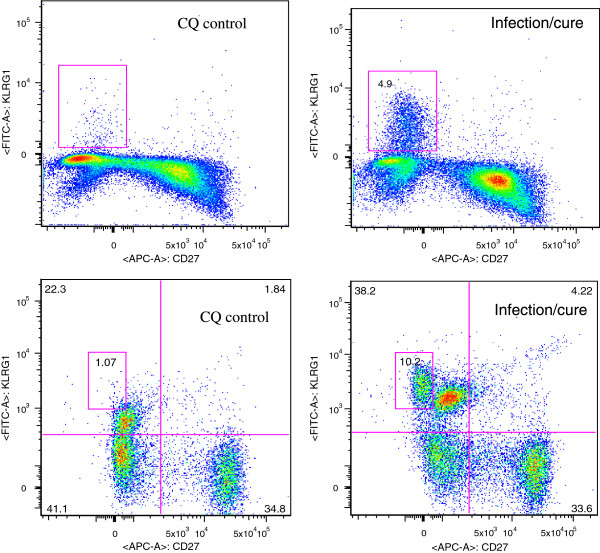
**Identification of CD8+ T cells phenotype generated during the infection/cure.** Splenocytes (top panels) or peripheral blood (bottom panels) from naïve (left panels) or infection/cure (right panel) animals were harvested three weeks post last inoculation. Total lymphocytes were extracted, and stained as described in methods section. Total lymphocytes, were gated on CD8+ T cells and analysed for the presence of KLRG1, and CD27.

**Figure 4:**
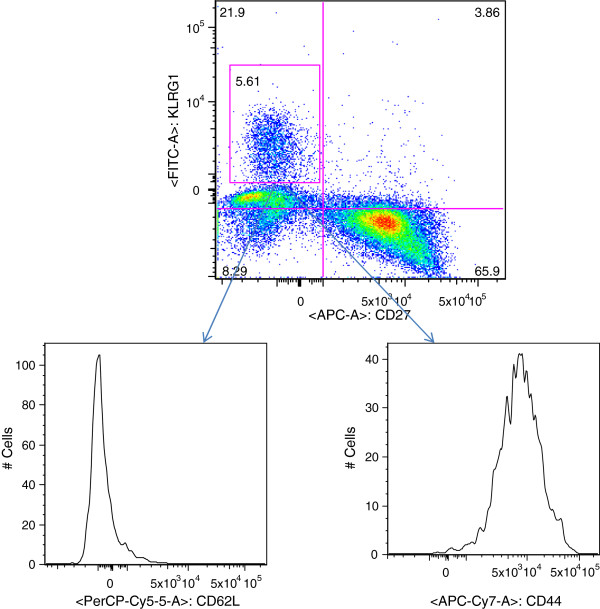
**Identification of CD8+ T cells phenotype generated during the infection/cure.** Splenocytes from infection/cure animals were gated on CD8+ KLRG1^high^, CD27^low^ cells, and analysed for the level of expression of CD62L and CD44 experiments which were representative of 3 experiments with five mice in each group.

In order to identify the phenotype of CD8+ T cells generated during this infection/cure under CQ cover regimen, splenocytes and peripheral blood from mice three weeks post last inoculation for the presence of CD8+, CD3+, CD11a, CD127, CD27, KLRG1, CD44 and CD62L cells by flow cytometry was analysed. Circulating peripheral lymphocytes of infection/cure mice had about six-fold higher percent of CD8+ T cells containing KLRG1^high^, CD27^low^ compared to controls (Table 2) with p value of control group vs. infection/cure group at <0.0001. Figure 3 shows a representative experiment where splenocytes and peripheral blood lymphocytes were analysed. Analysis of the CD8+ KLRG1^high^, CD27^low^ cell population had high expression of CD44 and low expression of CD62L (Figure 4). Infection/cure under CQ cover was thus associated with increased populations of effector memory CD8+ T cells. Results in Figures 3 and 4 are representative of three experiments with a pool of three spleens (splenocytes) or three experiments with 5–7 animals per group for the peripheral blood lymphocytes (peripheral blood). Statistical analysis for peripheral blood is reported in Table 2.

**Table 2:** **CD8+ T cells containing KLRG1**^**high**^**, CD27**^**low **^**phenotype**

**Group**	**Mean CD8+ KLRG1**^**high**^**, CD27**^**low **^**cells (%)**	**95% CI of mean**		**Number of lymphocytes/ml × 10^3**	**Absolute number of CD + KLRG1**^**high**^**, CD27**^**low **^**T cells × 10^3/ml of blood**
		Lower	Upper		
Control (15)	1.04	0.73	1.36	15,409	160.168
Post-3 (22)	5.72	4.41	7.04	14,894	841.32
Post-1 (5)	1.30	0.95	1.64	16,518	214.147

Infection/cure animals were inoculated three times (post-3) or one time with 20,000 Spz (post-1). The second column shows means of percent CD8+ cells expressing KLRG1^high^, CD27^low^ phenotype in the circulating peripheral lymphocytes. Statistical significance was determined using Mann Whitney test. The numbers of animals used in each experiment per group are shown in brackets. There was significant difference between the control vs. post-3 groups (p < 0.0001) and between post-3 vs. post-1 (p = 0.0013). There was no significant difference between control vs. post-1 groups. The last two columns show the number of CD8+ KLRG1^high^, CD27^low^ T cell × 10^3/ml of blood.

### Identifying T cell phenotype associated with protection

To investigate whether the phenotype CD8+ KLRG1^high^, CD27^low^, CD44^high^, CD62L^low^ is associated with protection, partial protection conferred by a two immunization regimen with 20,000 Spz (Table 1) was used. Twelve C57BL/6 mice were immunized twice with 20,000 Spz. For controls, three animals were injected with CQ only. Two weeks after suspension of CQ treatment, circulating peripheral blood was collected from individual mice. The presence of CD8+ KLRG1^high^, CD27^low^ cells was analysed. Animals were challenged and the appearance of parasitaemia monitored for two weeks. Expression of KLRG1^high^, CD27^low^ was significantly higher in protected animals compared to non-protected or controls with a p-value of 0.0087 and 0.024 respectively (Table 3), suggesting that CD8+, KLRG1^high^, CD27^low^, CD44^high^, CD62L^low^ cells were associated with protection.

**Table 3:** **Mean CD8+ KLRG1**^**high**^**, CD27**^**low **^**T cell (%) expressed in circulating peripheral blood of control, protected and non-protected animals**

**Group**	**Mean CD8+ KLRG1**^**high**^**, CD27**^**low **^**cells (%)**	**95% CI of mean**	
		Lower	Upper
Control (3)	1.117	0.3696	1.864
Protected(6)	5.772	3.759	7.785
Non-protected (6)	2.385	1.563	3.207

Twelve C57BL/6 mice were immunized twice with 20,000 Spz. Six mice were protected and six were not protected. Statistical significance was determined using Mann Whitney test. The numbers of animals used in each group are shown in brackets. There was significant difference between the control vs. protected groups (p = 0.024) and protected vs. non-protected (p = 0.0087). There was a marginal significant difference between control vs. non-protected (p = 0.0476).

## Discussion

Little progress has been made in elucidating the requirement for immune protection against malaria infection. Protection has not been achieved even with high titers of antibodies against known liver antigen [33], blood stage antigen [34,35] or antigen specific CD8+ T cells produced with adenovirus immunization [36]. In addition, human immunization with irradiated sporozoites has shown limited success unless sporozoites are injected intravenously [37]. Conversely, immunization with irradiated sporozoites or live sporozoites under CQ cover induces protection [29,31]. In the current study, the infection/cure C57BL/6 mouse model was used to elucidate the immune protection conferred during malaria infection by characterizing the nature of CD8+ T cells. Recent studies showed that both CD4+ T cells and CD8+ T cells are necessary to mediate immunity to liver stage malaria parasites [31,32]. This was demonstrated by immunizing BALB/c mice with *Plasmodium yoelii* iRBC under CQ cover and then treating with depleting antibodies against CD4+ T cells and CD8+ T cells. In this study, the infection/cure CD8+ T cells deficient (MHC I KO) mice resulted in only partial protection from lethal challenge, thus implicating CD8+ T cells in protection. Passive transfer with serum or cell alone also failed to confer 100% protection, thus indicating the cooperation of antigen specific cells and antibodies in granting protection. MHC II animals were also only partially protected. The most likely explanation is that the protective antibodies generated during infection/cure need CD4+ T cell help, which is lost in CD4+ T cell deficient animals. However, the role of CD4+ T cell in this model cannot be excluded is also to support the generation of protective CD8+ T cells or that they are directly involved in parasite killing. It will be interesting to perform transfer experiments to test the protective effect of serum from infection/cure MHC II animals enriched with CD4+ T cells from infection/cure wildtype animals. This will be important to discriminate between the roles of CD4+ T cells as pure ‘helper’ for antibodies production or more direct involved in anti-plasmodium activity. From this study and that by Belnoue *et al.*, data strongly suggest that both a humoral response and CD8+ T cells are required for immunity to liver stage *P. berghei* parasites [31,32].

In an effort to further elucidate immune protection, CD8+ T cells in peripheral blood and splenocytes were characterized. The distinct populations of CD8+ T cells with phenotype KLRG1^high^, CD27^low^, CD44^high^, CD62L^low^ were shown to be associated with protection. This population is absent in the intrahepatic lymphocytes. In the classical model of protection by immunization with irradiated sporozoites, the putative protective CD8+ T cells are found mostly in the liver [18,19] and produce IFNγ. In a recent study, Nganou-Makandop *et al.*[21] reported comparable levels of hepatic CD8+ T cells with a CD44^high^, CD62L^low^ phenotype that produce IFNγ in response to PMA/Iono. In this study, mice were either immunized with radiation-attenuated sporozoites or infected with *P. berghei* under CQ cover. In the current study, the CD8+ KLRG1^high^, CD27^low^, CD44^high^, CD62L^low^ phenotype that was associated with protection was not present in the liver of immunized animals. A possible explanation of the apparent discrepancy is that more than one population of CD8+ T cells contributes to protection. One can speculate that the splenic CD8+ KLRG1^high^, CD27^low^, CD44^high^, CD62L^low^ cells and the intrahepatic CD8+ CD44^high^, CD62L^low^ cells are the same antigen specific population whose phenotype and organ-homing is modulated by antigen exposure. In explorative experiments, it was observed that splenic and peripheral blood CD8+ KLRG1^high^, CD27^low^, CD44^high^, CD62L^low^ T cells respond to *in vitro* stimulation with anti CD3 and *in vivo* sporozoites infection by producing IFNg (see Additional file 1: Figure S1 and Figure S2). This is consistent with CD8+ KLRG1^high^, CD27^low^, CD44^high^, CD62L^low^ T cells participating to protection by producing IFNg. However, these cells do not account for all the IFNg produced in response to *in vitro* stimulation with anti CD3 (see Additional file 1: Figure S1 and Figure S2), or *in vivo* stimulation with sporozoites see Additional file 1: Figure S1 and Figure S2). It is, therefore, conceivable that other CD8+ T cells with low expression of KLRG1 are generated during this infection/cure regimen. Such cells respond to sporozoite infection with IFNg production and display an elevated ability to respond to T cell receptor triggered by releasing IFNg. The role of this CD8+ T cell phenotype in protection remains to be determined. KLRG1 has been shown to down regulate T cell receptor signaling [38,39], thus suggesting that the acquisition of the KLRG1^high^ phenotype is important for effector memory population after antigen encounter and execution of effector function.

## Conclusions

KLRG1^high^, CD27^low^, CD44^high^, CD62L^low^ are the circulating CD8+ T cells generated during infection which are associated with protection and thus represent a correlate of protection. Passive transfer of purified/enriched KLRG1^high^, CD27^low^, CD44^high^, CD62L^low^ CD8+ T cells will be critical in demonstrating a direct correlation of this phenotype in protection.

## Competing interests

The authors declare that they have no competing interests.

## Authors’ contributions

Conceived and designed the experiments: EK, CB, CFO. Performed the experiments: CB, EK. Analysed the data: CB, EK. Provided reagents and support: CFO, JM. Wrote the Manuscript: EK, CB. Reviewed the Manuscript: CFO, JHR. All authors approved the final version of the manuscript.

## Supplementary Material

Additional file 1 **Interferon produced by CD8+ KLRG1**^**high**^**, CD27**^**low**^**.** Infection/cure animals were challenged with 1,000 sporozoites three weeks post third immunization. Twenty four hours later, animals were injected with 100 μg of Brefeldine A to prevent lymphokines secretion. Five hours later animals were euthanized and blood collected by cardiac puncture. Five naïve mice were used as controls. Blood from 5 naïve or 5 infection/cure animals was pooled together, red blood cells were lysed and the peripheral lymphocytes incubated in the presence of media control of 5 μg/ml of anti CD3 antibody 2C11. Sixteen hours later cells were washed and stained for CD3, CD8, KLRG1, CD27 and IFNγ. Figure S1) Peripheral blood lymphocytes were gated on CD8+ KLRG1^high^, CD27^low^ and analyzed for IFNγ produced by CD8+ KLRG1^high^, CD27^low^. Figure S2) Splenocytes from animal infection/cure were labeled with CFSE, transferred into naïve mice and then challenged with 1,000 Spz 24hr post transfer. Forty-eight hours post challenge animals were euthanized and splenocytes harvested and analyzed for the production of IFNγ by transferred (immune cells) and recipient’s (naïve) cells. CFSE+ (transferred/immune) or CFSE- (naïve) cells were gated on CD8+, CD27^low^ and analyzed for KLRG1 and IFNγ. Click here for file
