# Supplementary material for: Phenotypic characterization of Plasmodium berghei responsive CD8+ T cells after immunization with live sporozoites under chloroquine cover
Source: Malar J. 2014 Mar 12;13:92. doi: 10.1186/1475-2875-13-92 (PMC4008132; doi:10.1186/1475-2875-13-92)
Supplement: Additional file 1 — Interferon produced by CD8+ KLRG1high, CD27low. Infection/cure animals were challenged with 1,000 sporozoites three weeks post third immunization. Twenty four hours later, animals were injected with 100 μg of Brefeldine A to prevent lymphokines secretion. Five hours later animals were euthanized and blood collected by cardiac puncture. Five naïve mice were used as controls. Blood from 5 naïve or 5 infection/cure animals was pooled together, red blood cells were lysed and the peripheral lymphocytes incubated in the presence of media control of 5 μg/ml of anti CD3 antibody 2C11. Sixteen hours later cells were washed and stained for CD3, CD8, KLRG1, CD27 and IFNγ. Figure S1) Peripheral blood lymphocytes were gated on CD8+ KLRG1high, CD27low and analyzed for IFNγ produced by CD8+ KLRG1high, CD27low. Figure S2) Splenocytes from animal infection/cure were labeled with CFSE, transferred into naïve mice and then challenged with 1,000 Spz 24hr post transfer. Forty-eight hours post challenge animals were euthanized and splenocytes harvested and analyzed for the production of IFNγ by transferred (immune cells) and recipient’s (naïve) cells. CFSE+ (transferred/immune) or CFSE- (naïve) cells were gated on CD8+, CD27low and analyzed for KLRG1 and IFNγ. [file 1475-2875-13-92-S1.pptx]

## Slide 1
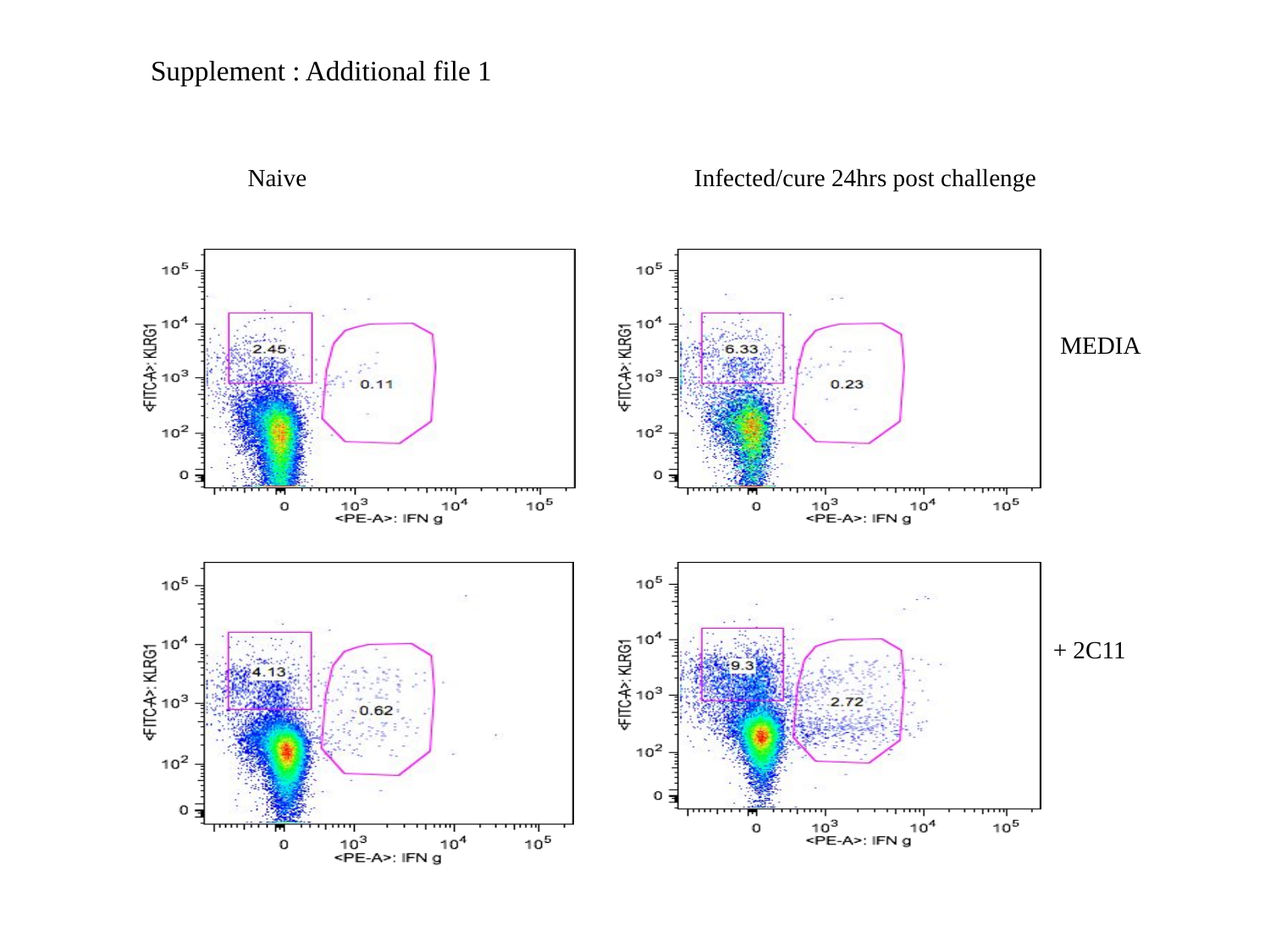

Supplement : Additional file 1
Naive
Infected/cure 24hrs post challenge
 MEDIA
+ 2C11

## Slide 2
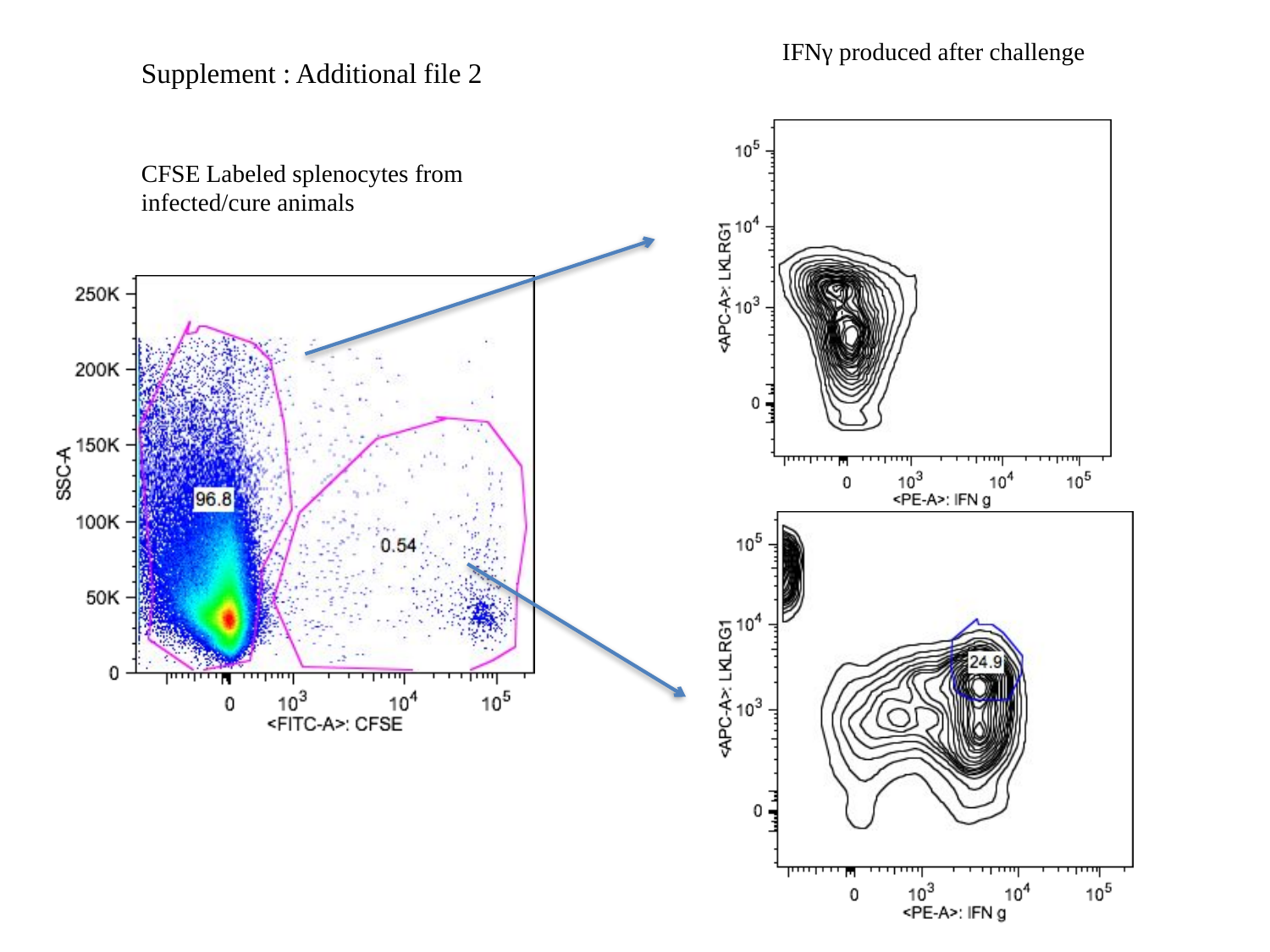

IFNγ produced after challenge
Supplement : Additional file 2
CFSE Labeled splenocytes from infected/cure animals
